# Anterior Cruciate Ligament Reconstruction Failure: Etiology, Classification, and Revision Strategies—A Narrative Review

**DOI:** 10.3390/jfmk11010077

**Published:** 2026-02-14

**Authors:** Giacomo Capece, Rosario Junior Sagliocco, Guido Bocchino, Andrea De Fazio, Emidio Di Gialleonardo, Alessandro El Motassime, Davide Messina, Agostino Fernicola, Giulio Maccauro, Raffaele Vitiello

**Affiliations:** 1 U.O.C. Orthopedics and Traumatology, Ospedale dei Pellegrini, 80134 Naples, Italy; dott.rosariojuniorsagliocco@gmail.com; 2 Orthopaedics and Trauma Surgery Unit, Department of Ageing, Neurosciences, Head-Neck and Orthopaedics Sciences, Fondazione Policlinico Universitario Agostino Gemelli, IRCCS, Largo A. Gemelli, 8, 00168 Rome, Italy; guido.bocchino01@icatt.it (G.B.); andrea.defazio01@icatt.it (A.D.F.); emidiodiggia@gmail.com (E.D.G.); alessandroelmotassime@gmail.com (A.E.M.); davide.messina01@icatt.it (D.M.); giulio.maccauro@unicatt.it (G.M.); 3 Orthopaedics and Trauma Surgery Unit, Catholic University of The Sacred Heart, 00168 Rome, Italy; 4 Department of Orthopaedic Surgery, University of Florence, 50134 Florence, Italy; 5 Unit of Emergency Surgery, Department of Advanced Biomedical Sciences, University of Naples Federico II, 80134 Naples, Italy; agostino.fernicola@unina.it

**Keywords:** ACL reconstruction, graft failure, revision surgery, tunnel malposition, biomechanical factors, neuromuscular control, functional outcomes

## Abstract

Anterior cruciate ligament (ACL) reconstruction is a common orthopedic procedure, but graft failure remains a significant complication, particularly in young and active individuals. Understanding the multifactorial etiology of failure and optimizing revision strategies are crucial for improving outcomes. A structured narrative review of the literature was conducted, including studies published from January 2000 to May 2024. Databases searched included PubMed/MEDLINE, Embase, and Google Scholar. Eligible studies addressed definitions, etiology, classification, and surgical management of ACL reconstruction failure. Data were synthesized qualitatively, integrating evidence on technical, biological, and traumatic causes, as well as neuromuscular and psychosocial factors influencing functional outcomes. ACL reconstruction failure is primarily caused by technical errors, particularly tunnel malposition (60–70% of cases), followed by traumatic (15–25%) and biological (10–15%) mechanisms. Failure timing provides diagnostic clues: early (<3 months) failures often relate to fixation or infection, mid-term (3–12 months) to technical errors, and late (>12 months) to trauma or degeneration. Revision strategies include individualized graft selection, anatomical tunnel placement, repair of associated lesions, and consideration of biomechanical abnormalities. Overall success rates of revision procedures average 70–75%, with lower outcomes in adolescents and high-demand athletes. Emerging techniques, including lateral extra-articular tenodesis and biologic augmentation, may enhance revision outcomes, although long-term evidence remains limited. ACL reconstruction failure is a multifactorial event requiring thorough preoperative assessment, precise surgical planning, and individualized management. Addressing technical, biological, and neuromuscular factors, alongside patient-specific considerations, is essential to optimize functional outcomes and reduce failure rates. Future research should focus on standardized reporting, multicenter prospective studies, and advanced surgical planning tools to further improve revision success.

## 1. Introduction

Anterior cruciate ligament (ACL) rupture is one of the most frequent injuries in young athletes, accounting for up to 50% of all ligamentous knee injuries and leading to significant socioeconomic impact due to time loss from sport and work [1]. ACL reconstruction has become a routine and highly standardized procedure in orthopedic sports medicine, with over 200,000 surgeries performed annually in the United States alone [2]. Despite advances in surgical techniques and rehabilitation protocols, graft failure remains a challenging complication, affecting between 1.8% and 16.7% of patients depending on follow-up duration and population characteristics [3].

Anterior cruciate ligament (ACL) reconstruction is a widely performed procedure in orthopedic sports medicine, particularly in young and physically active patients [4]. Despite generally positive outcomes, graft failure is a notable complication, with rates ranging from 1.8% to 16.7% depending on follow-up duration. Failure manifests through a combination of objective signs, such as positive Lachman and pivot-shift tests, instrumented laxity > 3 mm, and reduced knee range of motion, and subjective symptoms including pain, instability, or functional impairment [5]. Traditional definitions of failure focusing solely on mechanical instability are limited. Studies show that a significant portion of patients with positive laxity tests report satisfactory subjective outcomes, while others experience symptoms despite normal objective findings [6]. The 2022 ESSKA consensus defines ACL failure as abnormal knee function due to graft insufficiency or failure to restore expected knee stability, caused by trauma, microtrauma, technical errors, biological factors, or untreated associated lesions [7]. This broadened definition underscores that ACL reconstruction outcomes are multifactorial, involving both mechanical and biological determinants of stability.

Beyond mechanical stability, recent literature emphasizes the concept of functional knee stability, which depends not only on passive structures such as the graft and capsule but also on neuromuscular control and proprioceptive feedback mechanisms. Disruption of this sensorimotor integration may lead to subjective instability even when objective graft integrity is preserved, highlighting the complex interaction between biomechanical and neurophysiological recovery following ACL reconstruction.

Emerging evidence suggests that neuromuscular retraining and proprioceptive rehabilitation are essential to restore dynamic joint control and prevent recurrent instability [8].

In addition, the biopsychosocial dimension of postoperative recovery has gained increasing attention. Psychological readiness, fear of re-injury, and patient expectations have been shown to significantly influence subjective perception of function and return-to-sport rates [9]. These factors contribute to discrepancies between clinical tests and patient-reported outcomes, underscoring the need for a holistic assessment of surgical success.

Revision surgery success rates average around 75%, though outcomes vary by patient subgroup. Adolescents show higher failure rates (9–21%) and reduced return to pre-injury sport levels (27–68%). Primary ACL reconstruction failures typically range from 5% to 10% [10]. Recent large-scale registries and meta-analyses have confirmed that revision ACL reconstruction outcomes are consistently inferior to primary procedures, with higher rates of graft re-rupture and persistent instability [11]. These findings highlight the importance of preventing failure through meticulous surgical planning and comprehensive rehabilitation.

Etiologically, failure stems from recurrent instability (most common), postoperative complications (e.g., stiffness, infection), and patient factors (muscle dysfunction, arthritis). Technical errors, especially tunnel malposition, account for up to 80% of femoral and 37% of tibial tunnel-related failures. Biological failure involves inadequate graft integration, and traumatic failures often occur within the first postoperative year when biological incorporation is incomplete [12].

Failure timing offers diagnostic clues: early (<3 months) failures relate to fixation or infection, mid-term (3–12 months) to technical issues or aggressive rehab, and late (>12 months) to new trauma or degeneration. Infections are rare (<1%) but serious, requiring infection eradication before revision [13].

Late failures are increasingly recognized as multifactorial, often involving degenerative joint changes, secondary meniscal tears, or altered kinematics due to chronic instability [14].

Revision ACL reconstruction represents a growing clinical and research challenge. Despite numerous studies, consensus on optimal revision strategies remains limited, and current trends emphasize individualized approaches based on patient age, activity level, graft type, and associated injuries [15].

This review aims to provide an overview of ACL reconstruction failure classifications, causes, and current revision strategies, integrating recent perspectives on neuromuscular, biological, and psychosocial determinants of postoperative outcomes.

## 2. Materials and Methods

This work was designed as a narrative review. PRISMA 2020 principles were referenced to improve transparency and traceability of the literature search and reporting process [16]. The review protocol was prospectively registered in the PROSPERO database (registration number: 1170825).

A comprehensive literature search was carried out across the PubMed/MEDLINE, Embase, and Google Scholar databases to identify relevant publications from January 2000 to May 2024. The search strategy combined the following terms and Boolean operators: “anterior cruciate ligament reconstruction” OR “ACL reconstruction” AND “failure” OR “graft failure” OR “revision” OR “instability” OR “technical error” OR “tunnel malposition” OR “biological failure” OR “revision outcomes.” This strategy was designed to maximize sensitivity while maintaining specificity. In addition, the reference lists of the included studies and relevant reviews were manually screened to identify further eligible papers not retrieved during the initial database search.

Only articles published in English were considered eligible. Studies were included if they addressed at least one of the following topics: definitions and classifications of anterior cruciate ligament (ACL) reconstruction failure, etiological factors such as technical, biological, or traumatic causes, or clinical and surgical management of revision ACL reconstruction. Eligible study designs included original research, systematic and narrative reviews, consensus statements, and large multicenter cohort studies, with particular attention to contributions from the Multicenter ACL Revision Study (MARS) group. Exclusion criteria comprised case reports, letters, editorials, conference abstracts, and studies not directly related to ACL reconstruction failure.

Two reviewers (G.C. and R.S.) independently screened all retrieved titles and abstracts, followed by full-text assessment of potentially relevant studies. Disagreements were resolved through discussion with a senior author (R.V.) to ensure consensus and consistency. For each included study, data were extracted regarding study design, population characteristics, mechanisms and classifications of failure, surgical techniques, graft selection, and clinical outcomes following revision procedures.

Given the narrative nature of the review, no formal quantitative synthesis or meta-analysis was performed. However, the methodological quality of each study was qualitatively assessed based on clarity of design, adequacy of clinical data, and relevance to the review’s objectives. The extracted evidence was synthesized thematically, focusing on the etiology, classification, and management of ACL reconstruction failure.

**Figure 1:**
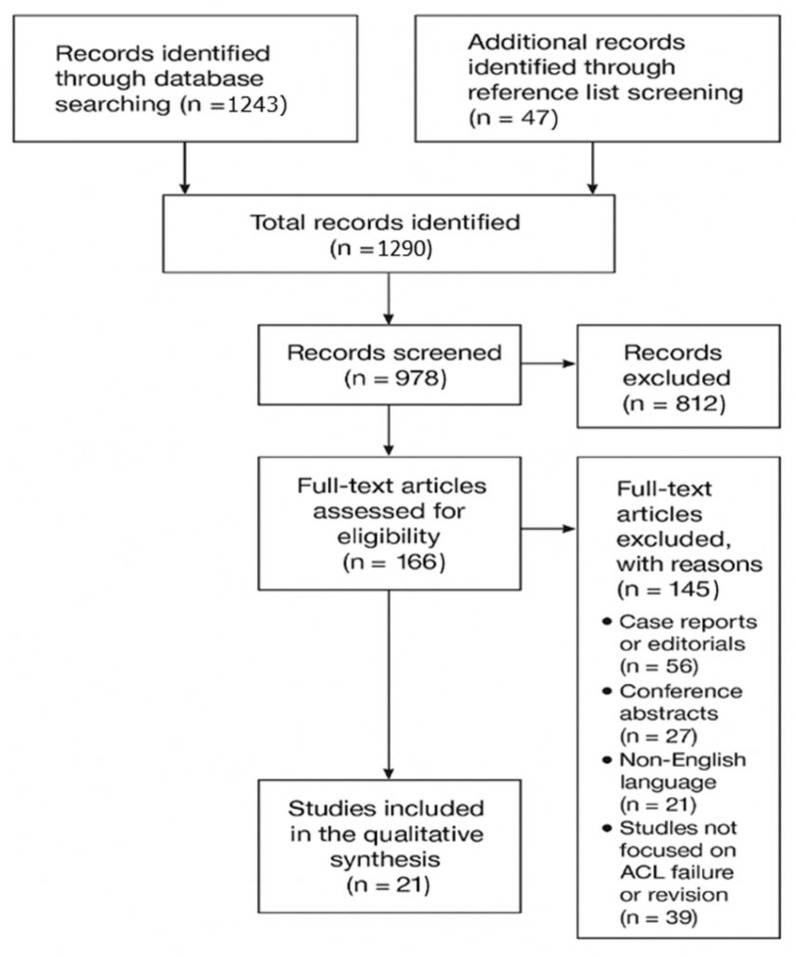
PRISMA Flow-Diagram.

The final synthesis integrated evidence-based data, expert consensus, and clinical experience to provide a comprehensive overview of current knowledge in the field of ACL reconstruction failure and revision surgery. The process of study selection is summarized in the PRISMA flow diagram (Figure 1), while the inclusion and exclusion criteria are presented in Table 1.

## 3. Results

### 3.1. Etiology of ACL Reconstruction Failure

ACL reconstruction failure is a multifactorial event involving technical, biological, and traumatic mechanisms. Among these, technical errors remain predominant, representing approximately 60–70% of all failures [Table 2].

The most common technical issue is femoral tunnel malposition, which is reported in up to 80% of failed reconstructions, typically due to an excessively anterior tunnel compromising graft isometry and tension balance. Posterior malpositioning of the tibial tunnel occurs in approximately 37% of cases and can lead to impingement and loss of stability. Other frequent causes include inappropriate graft selection, improper fixation or tensioning, and unaddressed concomitant lesions, such as meniscal ramp tears, anterolateral ligament (ALL) or posterolateral corner (PLC) injuries, that compromise rotational control and predispose to residual instability [Table 2]. Traumatic causes, responsible for about 15–25% of failures, often involve high-energy injuries during the first postoperative year, when biological incorporation is still incomplete. However, secondary traumatic ruptures may also occur years later due to new pivoting injuries or a return to contact sports. Biological failure, reported in 10–15% of cases, remains less clearly understood. It involves insufficient graft osteointegration, delayed revascularization, or incomplete ligamentization, often presenting without a distinct traumatic episode or technical fault. MRI and second-look arthroscopy studies have demonstrated variable graft remodeling and vascular ingrowth within the first 12–24 months postoperatively, with incomplete maturation associated with inferior mechanical strength and higher re-tear risk. Postoperative complications, such as infection (<1%), hematoma, and arthrofibrosis, may further compromise outcomes. Moreover, patient-related factors (smoking, diabetes, low quadriceps strength, high BMI, or premature return to sport) have been identified as independent predictors of revision surgery [Table 3].

### 3.2. Classification of Failure

ACL reconstruction failures can be classified according to their etiology (technical, biological, or traumatic) or timing (early, mid-term, or late), as illustrated in Table 4.

Early failures (<3 months) are typically linked to fixation loss, infection, or acute trauma; mid-term failures (3–12 months) are commonly associated with technical errors, tunnel widening, or overly aggressive rehabilitation; and late failures (>12 months) often result from new trauma, progressive graft elongation, or degenerative changes.

More refined classification systems, such as the Pittsburgh group or MARS criteria, integrate both failure mode and timing, aiding surgical planning by correlating the cause of failure with the most appropriate revision approach. In addition, the concept of “functional failure” has emerged to describe cases with persistent instability despite anatomically correct reconstruction, highlighting the influence of neuromuscular and sensorimotor deficits on knee kinematics.

### 3.3. Treatment and Surgical Strategies

Revision ACL reconstruction requires a personalized, stepwise approach based on the etiology of failure, tunnel condition, graft choice, and presence of associated lesions. Overall, the success rate after revision surgery averages 70–80%, though outcomes remain inferior to primary reconstructions, particularly in adolescents, where failure rates can reach 35% and return-to-sport rates remain below 65% [Table 3]. Preoperative assessment should include 3D imaging (CT or MRI) to evaluate tunnel position, enlargement, and bone stock. Significant tunnel widening (>13 mm femoral or >16 mm tibial) generally necessitates a two-stage revision, with bone grafting and subsequent healing verification (typically 20–24 weeks post-grafting). Graft selection is a key determinant of outcome [Table 5].

Bone–patellar tendon–bone (BPTB) autografts remain the gold standard due to reliable bone-to-bone healing and fixation strength, especially when reusing previous tunnels. Quadriceps tendon grafts have gained popularity for their low donor-site morbidity and consistent cross-sectional area, offering versatility in complex revisions. Hamstring autografts, though strong, show higher elongation rates when tunnels are dilated or fixation is suboptimal. Allografts may be considered in older or low-demand patients but carry higher failure rates in young, active individuals, up to three times greater according to MARS data. Fixation and tensioning are critical: optimal tension ranges between 20–40 N, with the knee positioned at 20–30° of flexion to ensure balanced anterior–posterior and rotational stability. Anatomical tunnel placement is guided by the Bernard–Hertel quadrant method (femur) and Tsukada’s landmarks (tibia). When anatomical constraints preclude standard positioning, alternative techniques such as out-in femoral drilling, over-the-top reconstructions, or lateral extra-articular tenodesis (LET) may be indicated to restore rotational stability. Addressing associated lesions is paramount: unrecognized ALL injuries, PLC insufficiency, or meniscal ramp tears are implicated in up to 25–30% of revision cases and should be systematically repaired. Furthermore, biomechanical abnormalities such as an increased posterior tibial slope (>12°) significantly elevate anterior tibial translation forces and may justify a corrective osteotomy to reduce graft stress.

Recent literature has also emphasized the potential benefit of augmented reconstructions, including the use of synthetic internal braces or biological adjuncts (platelet-rich plasma, stem-cell scaffolds) to enhance graft maturation and reduce re-tear risk, though long-term data remain limited.

To improve clarity and provide a concise overview of the main determinants of ACL reconstruction failure, a summary table synthesizing key evidence and related surgical implications is presented in Table 6.

**Figure 2:**
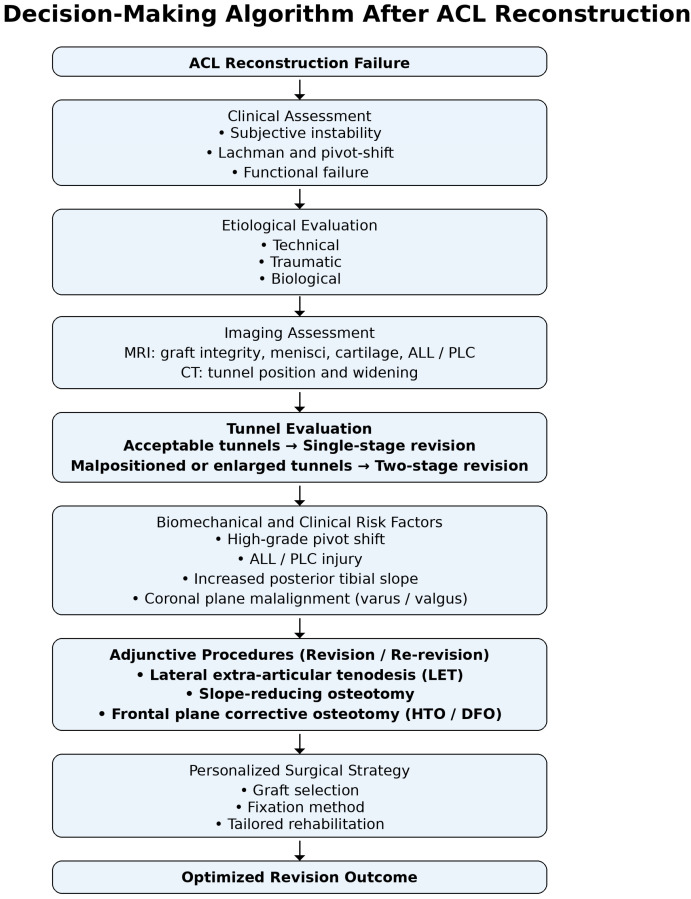
Decision-making flowchart for the management of anterior cruciate ligament (ACL) reconstruction failure. The algorithm summarizes clinical assessment, etiological evaluation, imaging-based tunnel analysis, and identification of biomechanical risk factors to guide surgical strategy. In revision and re-revision cases, adjunctive procedures such as lateral extra-articular tenodesis (LET) and corrective osteotomies are considered to optimize rotational stability and graft survivorship.

**Figure 3:**
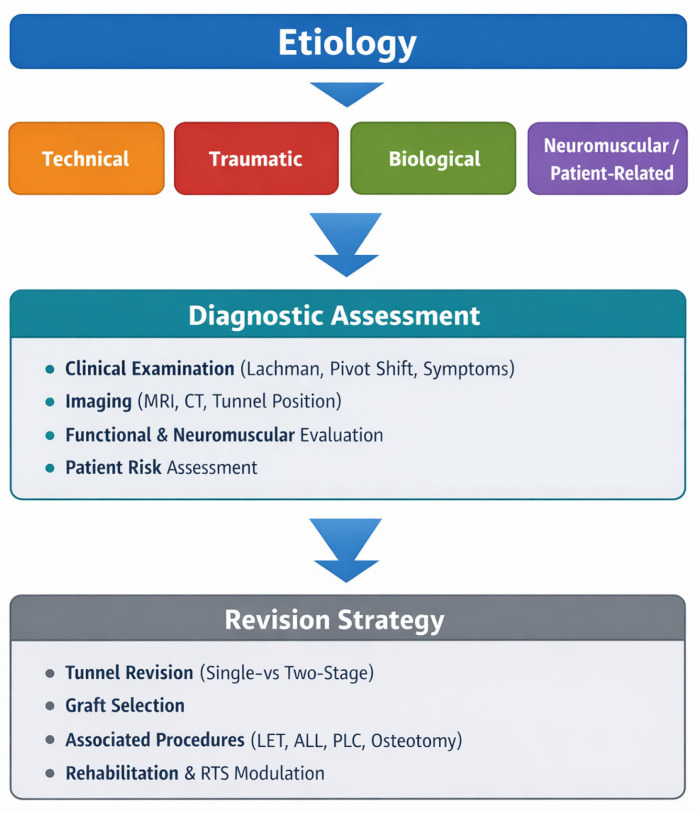
Conceptual framework linking etiology, diagnostic assessment, and revision strategy in ACL reconstruction failure.

Figure 2 summarizes a proposed decision-making algorithm for the management of ACL reconstruction failure, integrating etiological assessment, tunnel evaluation, and adjunctive surgical strategies. To further clarify the relationship between failure etiology, diagnostic work-up, and surgical decision-making, a conceptual framework is illustrated in Figure 3.

## 4. Discussion

Compared with primary ACL reconstruction, revision procedures are associated with greater technical complexity, higher rates of associated lesions and tunnel-related issues, and consistently inferior clinical outcomes, requiring more extensive preoperative assessment and individualized surgical planning [17]. The findings of this review confirm that technical errors, particularly tunnel malposition, remain the leading and largely preventable cause of graft failure, accounting for approximately 60–70% of cases, as consistently reported by large observational studies and registry-based analyses [18]. Anterior malpositioning of the femoral tunnel increases graft tension, predisposing to rupture, whereas posterior malposition of the tibial tunnel compromises joint kinematics and rotational stability. When tunnels are inadequately positioned or overlapping, two-stage revision procedures with bone grafting are often required, which prolong rehabilitation and increase patient morbidity [19].

The selection of an appropriate graft is central to surgical success. BPTB autografts provide reliable bone-to-bone healing and strong fixation, particularly advantageous when reusing previous tunnels [20]. Quadriceps tendon grafts offer versatility and low donor-site morbidity, while hamstring autografts may show weaker fixation, especially in the presence of tunnel enlargement [21,22]. Allografts, although convenient, carry higher failure rates in young, active patients, according to data derived from large multicenter registries, consistent with data from multicenter cohorts such as the MARS study [23]. Recent evidence further supports the use of quadriceps tendon autografts as a reliable alternative in revision settings, with comparable biomechanical strength to BPTB and superior patient-reported outcomes in some studies [24]. In contrast, the use of synthetic grafts has shown inconsistent results and remains controversial due to higher elongation rates and inflammatory complications [25]. These heterogeneous findings likely reflect differences in patient characteristics, tunnel morphology, fixation techniques, and follow-up duration. Consequently, graft selection should be guided by an individualized assessment of anatomical constraints, patient-specific risk factors, and surgeon expertise rather than by graft type alone. Outcomes after revision ACL reconstruction show considerable heterogeneity across patient populations, with age, activity level, and graft type acting as key modifiers of failure risk and functional recovery, particularly in young, high-demand athletes compared with older or lower-demand individuals.

Associated lesions, including meniscal ramp tears, anterolateral ligament (ALL), and posterolateral corner (PLC) injuries, are frequently underestimated but significantly impact rotational stability and revision outcomes [26]. Addressing these concomitant injuries is crucial to restore functional knee stability, as persistent neuromuscular deficits or uncorrected ligamentous insufficiency may result in subjective instability despite anatomically correct graft placement [27]. Moreover, biomechanical abnormalities such as increased posterior tibial slope or coronal malalignment may necessitate corrective osteotomy to optimize long-term outcomes [28]. Several recent studies, including meta-analyses and large observational cohorts, have confirmed that combined procedures, such as LET or ALL reconstruction, can significantly reduce rotational laxity and improve graft survivorship, particularly in high-risk populations such as young pivoting athletes [29,30].

Beyond structural and technical factors, neuromuscular control and psychological readiness are increasingly recognized as key modulators of functional outcomes after revision ACL reconstruction. Altered sensorimotor integration, impaired muscle activation patterns, and deficient proprioceptive feedback may contribute to persistent subjective instability and abnormal knee kinematics, even in the presence of an anatomically intact graft [31,32]. Although not all studies demonstrate a direct association between objective neuromuscular deficits and graft rupture, these factors appear to influence functional recovery, rehabilitation adherence, and return-to-sport success rather than structural failure alone, helping to explain discrepancies between objective stability measures and patient-reported outcomes.

From a clinical perspective, these findings support the use of individualized, criterion-based rehabilitation protocols that prioritize neuromuscular retraining, movement quality, proprioception, and load management over time-based progression alone. Psychological factors such as fear of re-injury, confidence, and readiness to return to sport should also be systematically addressed. Preoperative identification of neuromuscular and psychological risk factors may improve patient counseling and risk stratification, while postoperative multidisciplinary rehabilitation strategies integrating physical and psychological support may reduce recurrent instability and optimize long-term functional outcomes.

Technological advancements, including three-dimensional computed tomography (CT), virtual surgical planning, and techniques such as out-in femoral tunnels, over-the-top reconstructions, and selective double-bundle augmentation, have improved the precision of revision ACL reconstruction, enabling tailored approaches in complex anatomical scenarios [33,34]. Emerging strategies, such as LET and biologic augmentation with platelet-rich plasma or stem cells, may further reduce re-rupture risk; however, long-term evidence supporting their clinical effectiveness remains limited and controversial [35]. Although some studies report improved graft maturation and early functional recovery, others fail to demonstrate durable reductions in failure rates or long-term clinical advantages, with substantial heterogeneity in biologic agents, application protocols, and outcome measures limiting comparability across studies. In parallel, recent developments in intraoperative navigation, augmented reality, and robotic-assisted drilling have shown promising potential to improve tunnel placement accuracy and reduce technical variability among surgeons [36], although these technologies are not yet widely adopted.

Despite these advances, several important controversies and unresolved questions persist. Conflicting evidence remains regarding optimal graft choice across different revision scenarios, particularly concerning the relative benefits of BPTB, quadriceps tendon, and hamstring autografts. Similarly, indications for routine use of lateral extra-articular procedures remain debated, especially in low-risk or non-pivoting patients, where the balance between improved rotational stability and potential overconstraint is not clearly defined. The role of biologic augmentation also remains uncertain, as robust evidence demonstrating long-term reductions in failure rates or superior functional outcomes is still lacking. Furthermore, the relative contribution of neuromuscular and psychosocial factors to structural graft failure versus subjective instability remains incompletely understood, underscoring the need for standardized definitions of failure, homogeneous outcome reporting, and high-quality prospective studies.

Despite ongoing innovations, long-term outcomes after revision ACL reconstruction remain inferior to those of primary procedures, with reported success rates averaging 70–75% and reduced rates of return to pre-injury sport, particularly among adolescents and high-demand athletes. These findings highlight the multifactorial nature of ACL reconstruction failure, in which technical, biological, traumatic, and patient-specific factors interact to influence prognosis. Meta-analyses further confirm that psychological readiness, persistent muscle weakness, and residual instability are key determinants of unsuccessful return to sport [37]. Future research should therefore prioritize standardized failure definitions, integration of patient-reported outcome measures, and investigation of biological adjuvants to enhance graft integration and healing. Multidisciplinary approaches combining orthopedic, biomechanical, and psychological expertise are increasingly advocated to optimize functional recovery and patient satisfaction.

Re-revision ACL reconstruction represents a particularly complex clinical scenario, with outcomes consistently inferior to both primary and first-time revision procedures. Patients undergoing multiple revisions frequently present with residual rotational instability, compromised bone stock, tunnel enlargement, and uncorrected biomechanical risk factors, all of which increase the risk of further graft failure. Accordingly, re-revision surgery should be approached as a comprehensive corrective strategy rather than a repetition of standard revision techniques, addressing both intra-articular and extra-articular contributors to instability. Coronal plane malalignment should be systematically evaluated and corrected when indicated, as persistent varus or valgus alignment increases graft loading and predisposes to recurrent failure. In addition, lateral extra-articular tenodesis represents a valuable adjunct in re-revision settings—particularly in young patients, pivoting athletes, and cases with high-grade pivot shift—where it may improve rotational control and graft survivorship. Overall, available evidence supports a multimodal and individualized approach integrating anatomical graft placement, extra-articular augmentation, and corrective osteotomy when biomechanical risk factors are present to reduce the likelihood of repeated failure.

### Study Limitations

This narrative review has inherent limitations related to its methodological design. The absence of a systematic review protocol and of a quantitative meta-analysis precludes precise estimation of effect sizes and limits the ability to formally compare outcomes across studies. As study selection was based on qualitative synthesis, a degree of selection bias cannot be excluded, and the findings should be interpreted as descriptive rather than definitive.

In addition, the included studies are highly heterogeneous with respect to study design, patient populations, follow-up duration, surgical techniques, and definitions of ACL reconstruction failure, which further constrains direct comparability and limits the generalizability of conclusions. The relative scarcity of high-quality longitudinal, prospective, randomized trials in the field represents an additional limitation, reducing the strength of evidence supporting specific surgical or rehabilitative strategies.

These limitations imply that the conclusions of this review should be interpreted with caution and viewed as an integrative overview of current evidence rather than as practice-defining recommendations. Nevertheless, this review provides a comprehensive synthesis of available literature on ACL reconstruction failure, highlights consistent trends across studies, and identifies relevant gaps in knowledge. The findings underscore the need for standardized definitions of failure, homogeneous outcome reporting, and high-quality multicenter prospective studies to enable more robust quantitative analyses and evidence-based recommendations in the future.

## 5. Conclusions

ACL reconstruction failure is a multifactorial event that requires comprehensive assessment and individualized surgical planning. Successful revision surgery depends on technical precision in tunnel placement, appropriate graft selection, and thorough management of associated lesions, including meniscal, anterolateral, and posterolateral structures.

The inherent complexity of revision ACL procedures necessitates meticulous preoperative evaluation, supported by advanced imaging, detailed anatomical assessment, and careful consideration of patient-specific risk factors such as age, activity level, comorbidities, and neuromuscular status. Although revision outcomes are generally favorable, they remain inferior to primary reconstructions, with lower rates of return to pre-injury sport and increased risk of graft re-rupture.

Future advancements in this field will rely on the implementation of sophisticated surgical planning tools, precision-guided graft placement, and enhanced patient stratification. Moreover, ongoing clinical research, including longitudinal and multicenter studies, is essential to refine surgical strategies, improve graft integration, and optimize long-term functional outcomes, ultimately reducing failure rates in both primary and revision ACL reconstructions.

## 6. Practical Recommendations for Clinical Practice

○ Systematic evaluation of tunnel position and widening using MRI and/or CT is essential before revision ACL reconstruction.
○ Failure etiology should guide revision strategy; technical errors require anatomical tunnel correction, while traumatic failures require reassessment of return-to-sport timing.
○ Associated lesions (ALL, PLC, meniscal ramp tears) should be actively searched for and addressed to restore rotational stability.
○ Graft selection must be individualized according to tunnel morphology, patient age, and activity level rather than graft type alone.
○ Biomechanical risk factors (e.g., increased posterior tibial slope or malalignment) should be identified preoperatively and corrected when indicated.
○ Rehabilitation and return-to-sport decisions should follow criterion-based protocols, integrating neuromuscular and psychological assessment.

## Figures and Tables

**Table 1:** Inclusion and exclusion criteria.

Inclusion Criteria	Exclusion Criteria
Original research, narrative/systematic reviews, consensus statements, and multicenter studies on ACL reconstruction failure	Case reports, editorials, letters, conference abstracts
Studies addressing definitions, etiology (technical, biological, or traumatic), or surgical management of ACL failure	Studies not directly related to ACL reconstruction or revision
English-language publications (2000–2024)	Non-English publications

**Table 2:** Etiology of ACL Reconstruction Failure. Data derived from large observational studies, registry-based analyses, and systematic reviews [12]. Percentages are approximate and reflect ranges reported in the literature.

Etiology	Description	Approximate Frequency
Technical causes	Tunnel malposition (femoral anterior, tibial posterior), poor graft choice, fixation errors	60–70%
Traumatic causes	High-energy trauma, especially within first postoperative year	15–25%
Biological causes	Poor graft osteointegration, revascularization or ligamentization	10–15%

**Table 3:** Incidence and Risk Factors of ACL Reconstruction Failure. Incidence rates are derived from registry data, large cohort studies, and meta-analyses [10,11]. Reported values vary according to age, activity level, and follow-up duration.

Factor	Incidence/Rate	Notes
Overall failure rate	4–25%	Varies by demographics and activity level
Female patients (<20 or >40 years)	Higher incidence	Hormonal and biomechanical influences
Adolescents (<20 years)	Up to 35% failure	Highest risk subgroup; early return to sport
Revision surgery success rate	~75%	Lower in adolescents and high-level athletes
Early return to sport (<9 months)	↑ failure risk	Associated with incomplete graft maturation

**Table 4:** Timing-Based Classification of ACL Failure. Classification adapted from commonly used timing-based frameworks and consensus definitions, including ESSKA and MARS criteria [7,13].

Time from Surgery	Common Causes	Clinical Considerations
Early (<3 months)	Fixation failure, infection	Requires urgent management
Mid-term (3–12 months)	Technical errors, aggressive rehab, missed lesions	Surgical re-evaluation critical
Late (>12 months)	New trauma, graft elongation, degenerative widening	Assess for secondary instability

**Table 5:** Graft Options and Considerations in Revision ACL Reconstruction. Indications and considerations are based on evidence from comparative cohort studies, registry data, and expert consensus statements.

Graft Type	Advantages	Disadvantages	Indications
Bone–Patellar Tendon–Bone (BPTB)	Strong bone-to-bone healing, rigid fixation	Anterior knee pain, donor-site morbidity	Reuse of previous tunnels, high-demand athletes
Quadriceps Tendon	Versatile, low donor-site morbidity, large cross-section	Less widespread familiarity	Complex or revision cases
Hamstring Tendon	Good strength, minimal anterior knee pain	Weaker fixation in enlarged tunnels	Primary or single-stage revisions
Allograft	No donor morbidity, shorter operative time	Higher re-rupture rate in young patients	Older or low-demand individuals

**Table 6:** Key evidence summary of ACL reconstruction failure and implications for revision surgery.

Key Factor	Evidence from Literature	Clinical Relevance	Implications for Revision Strategy
Technical errors (tunnel malposition)	Most frequent cause of failure (≈60–70%); femoral tunnel malposition reported in up to 80% of failed ACL reconstructions	Persistent anterior and rotational instability	Anatomical tunnel repositioning; single- or two-stage revision depending on tunnel widening
Traumatic re-rupture	Accounts for 15–25% of failures, frequently occurring within the first postoperative year	Acute graft rupture after return to sport or high-energy trauma	New graft reconstruction; reassessment of return-to-sport timing and risk factors
Biological failure	Reported in 10–15% of cases; related to impaired graft integration or ligamentization	Progressive laxity without clear traumatic event	Optimization of graft choice and fixation; consideration of biological augmentation
Missed or untreated associated lesions (ALL, PLC, meniscal ramp tears)	Present in up to 25–30% of revision cases	Residual rotational instability despite intact graft	Concomitant repair or reconstruction (e.g., LET, ALL, PLC repair)
Patient-related risk factors (young age, early RTS, neuromuscular deficits)	Higher failure rates in adolescents and patients returning to sport < 9 months	Increased re-rupture risk and suboptimal functional outcomes	Individualized rehabilitation; delayed, criterion-based return to sport
Biomechanical abnormalities (e.g., increased posterior tibial slope)	Increased ACL graft loading associated with higher failure risk	Recurrent instability despite technically correct reconstruction	Consider corrective osteotomy in selected high-risk cases

## Data Availability

All the data we analysed and tables we compiled are available for any clarification.

## Abbreviations

ACL	Anterior Cruciate Ligament
ALL	Anterolateral Ligament
BPT	Bone–Patellar Tendon–Bone
CT	Computed Tomography
LET	Lateral Extra-articular Tenodesis
MRI	Magnetic Resonance Imaging
MARS	Multicenter ACL Revision Study
PLC	Posterolateral Corner
ROM	Range of Motion
ESSKA	European Society of Sports Traumatology, Knee Surgery & Arthroscopy
BMI	Body Mass Index
